# Efficient Capturing of Polycyclic Aromatic Micropollutants From Water Using Physically Crosslinked DNA Nanoparticles

**DOI:** 10.3389/fchem.2020.00002

**Published:** 2020-01-28

**Authors:** Siriki Atchimnaidu, Hari Veera Prasad Thelu, Devanathan Perumal, Kaloor S. Harikrishnan, Reji Varghese

**Affiliations:** School of Chemistry, Indian Institute of Science Education and Research (IISER) Thiruvananthapuram, Thiruvananthapuram, India

**Keywords:** DNA nanostructures, self-assembly, micropollutants, water purification, host-guest interactions

## Abstract

Design and synthesis of physically (non-covalently) cross-linked nanoparticles through host-guest interaction between β-CD and adamantane is reported. Specific molecular recognition between β-CD functionalized branched DNA nanostructures (host) and a star-shaped adamantyl-terminated 8-arm poly(ethylene glycol) polymer (guest) is explored for the design of the nanoparticles. The most remarkable structural features of DNA nanoparticles include their excellent biocompatibility and the possibility of various non-covalent interactions with both hydrophobic and hydrophilic organic molecules. Potential of DNA nanoparticles for the rapid and efficient capture of various micropollutants typically present in water including carcinogens (hydrophobic micropollutants), organic dyes (hydrophilic), and pharmaceutical molecules (hydrophilic) is also demonstrated. The capture of micropollutants by DNA nanoparticles is attributed to the various non-covalent interactions between DNA nanoparticles and the micropollutants. Our results clearly suggest that DNA based nanomaterials would be an ideal candidate for the capturing and removal of both hydrophilic and hydrophobic micropollutants typically present in water.

## Introduction

One of the major threats to human health is the occurrence of organic micropollutants such as pesticides, polycyclic aromatic hydrocarbons (carcinogens), pharmaceuticals etc. in drinking water, and hence their detection, capture, and removal from drinking water is extremely vital (Schwarzenbach et al., 2006). Micropollutants typically present in drinking water include both hydrophobic and hydrophilic organic molecules. Activated charcoal was traditionally used as sorbent for the capture and removal of micropollutants from drinking water (Órfão et al., 2006). Though activated charcoal is efficient and successful in removing hydrophobic micropollutants, they often fail in capturing the hydrophilic micropollutants. Nanomaterials offer an alternative and convenient substrate for the capture and removal of micropollutants from water. For example, carbon-based nanostructures such as multi-walled carbon nanotubes (Kuo, 2009), graphene (Xu et al., 2012), and graphene oxide (Gao et al., 2012) were proven to be remarkable adsorbents for organic micropollutants. In the case of carbonaceous nanomaterials, since the adsorption is mainly achieved through the π-π stacking interactions, they were shown to be very efficient for the capturing of polycyclic aromatic hydrocarbons. However, carbonaceous nanomaterials were found not to be a superior adsorbent for hydrophilic micropollutants. Similarly, metal nanoparticles were also found to be promising substrate for the removal of micropollutant from water (Wang et al., 2018). However, the toxicity associated with the metal nanoparticle is a concern. Very recently, supramolecular approaches were also reported for the capture of micropollutants. For example, hydrophobic cavity of β-cyclodextrin (β-CD) was explored for the rapid capturing of different organic micropollutants using a porous β-cyclodextrin-based polymer (Alsbaiee et al., 2016). In another report, charge-transfer interaction was used for the capturing of polycyclic aromatic hydrocarbons using a semi-rigid cationic cyclophane as the host (Barnes et al., 2012). Even though there are few elegant strategies are reported in the literature, there is always a growing demand for the design of new nanomaterials for the capturing of organic micropollutants that exhibit (i) excellent biocompatibility, (ii) efficient capturing (interaction) toward both hydrophobic and hydrophilic micropollutants, and (iii) non-toxicity.

The unique structural characteristics of DNA such as nanoscale dimensions, predictable secondary structure, ease of synthesis and molecular recognition-directed self-assembly have fascinated scientists to use DNA as a molecular building block for the construction of DNA nanostructures (Seeman, 1982, 2010; Gothelf and LaBean, 2005; Aldaye et al., 2008; Pinheiro et al., 2011; Tørring et al., 2011). Recent years have seen the emergence of extremely complex one-dimensional (1D), 2D, and 3D nanostructures made of DNA, which have found potential applications in various fields ranging from material science to medicine (Walsh et al., 2011; Jiang et al., 2012; Modi et al., 2013) to nanotechnology (Seeman, 2003; Feldkamp and Niemeyer, 2006; Song et al., 2013; Jones et al., 2015). More interestingly, DNA is known to have efficient non-covalent (physical) interactions with both hydrophilic and hydrophobic organic molecules through various non-covalent interactions such as hydrogen bonding, π-π stacking, electrostatic interactions etc. (Hannah and Armitage, 2004). Hydrophobic molecules prefers to bind to DNA through intercalation within the nucleobases through hydrophobic interactions. Whereas hydrophilic cationic molecules binds to the phosphate backbone of DNA through electrostatic interaction. The remarkable structural features of DNA including the excellent biocompatibility, efficient non-covalent interactions with hydrophobic and hydrophilic molecules and non-toxicity indisputably suggest that DNA based nanomaterials would be a promising candidate for the capture of organic micropollutants from water. Among the various classes of nanomaterials, nanogels, particularly physically cross-linked, are unique owing to their nanosize, porosity, and high guest encapsulation ability (Yu et al., 2006). Moreover, physically cross-linked nanogels are far superior over the chemically cross-linked counterparts because of their ease of synthesis, reversible and stimuli responsive nature (Mishra et al., 2018). Hence physically cross-linked DNA-based nanogel would be the most ideal candidate for the capture of organic micropollutants from water. Though chemically cross-linked DNA hydrogels have successfully explored for the removal of organic micropollutants from water, there is no report on the use of physically cross-linked DNA nanogels for the capture of micropollutants from water.

**Scheme 1:**
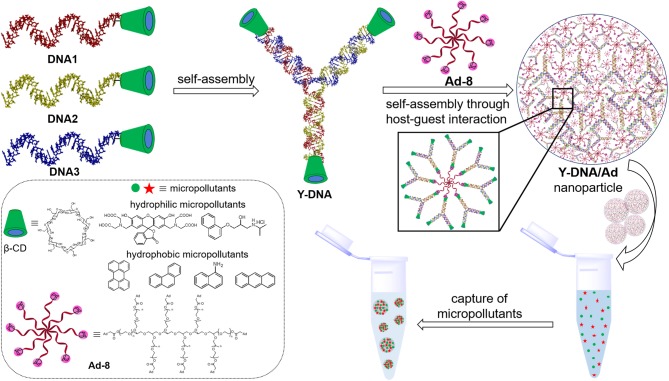
Pictorial representation showing the structures of **DNA1–3** and their self-assembly into β-CD tethered branched Y-DNA (**Y-DNA**). Self-assembly of **Y-DNA** with **Ad-8** through multivalent host–guest interactions between β-CD and adamantane to **Y-DNA/Ad-8** nanoparticle and the capture of hydrophilic and hydrophobic micropollutants by DNA nanogel are also shown. Chemical structures of β-CD, **Ad-8** and micropollutants investigated in this study are shown in the box.

Supramolecular interactions have been extensively explored for the design of various soft nanomaterials with remarkable optical and electronic properties (Rest et al., 2014; Taniguchi et al., 2016; Mishra et al., 2018; Zhou et al., 2018). Very recently, we have explored the molecular recognition between β-CD and adamantane for the design of DNA-based physically cross-linked nanogel (Thelu et al., 2018). We have demonstrated the synthesis of DNA-based nanogel with controllable size using the multivalent host–guest interaction between β-CD functionalized branched DNA nanostructures as the host and a star-shaped adamantyl-terminated 8-arm poly(ethylene glycol) polymer as the guest. The potential of the nanogel as a nanocarrier for targeted drug delivery was also demonstrated. Herein, we report the potential of DNA-based nanogel for the capture of organic micropollutants, both hydrophobic and hydrophilic, from water (Scheme 1). The design of nanogel was already reported by us, and the same strategy was followed for the synthesis of DNA nanogel. This was achieved by self-assembly between multivalent host–guest interaction between β-CD functionalized branched DNA nanostructures as the host and a star-shaped adamantyl-terminated 8-arm poly(ethylene glycol) (PEG) polymer as the guest. The size of the nanogel could be controlled by appropriate modulation of the concentration of the guest and host. The potential of the nanogel to capture a series of hydrophobic molecules and hydrophilic molecules are demonstrated. The capture of the molecules into the DNA nanogel network is due to the non-covalent interactions of the micropollutants with the backbone and bases of DNA. Our results clearly show that the DNA based nanogels are promising nanomaterials for the capture of micropollutants.

## Results and Discussions

**Figure 1:**
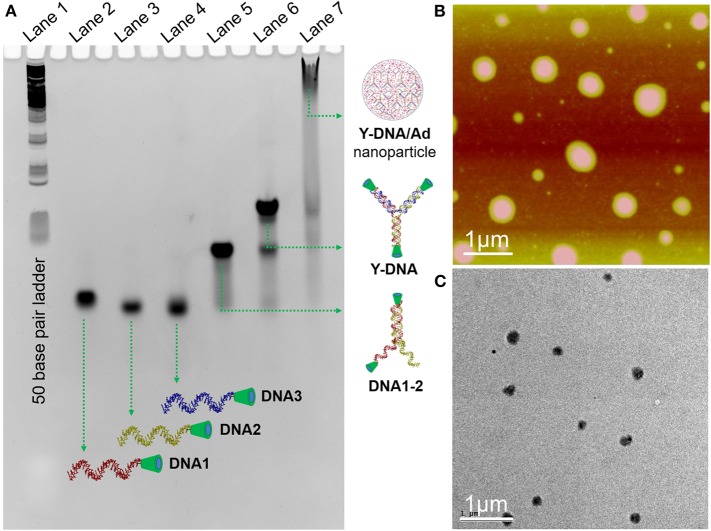
**(A)** Native PAGE (14%) analysis of the self-assembly of **Y-DNA/Ad-8**. L1 for 50 base pair ladder, L2–L4 for **DNA1–3**, respectively, L5 for the assembly of **DNA1** and **DNA2**, L6 for the assembly of **DNA1**, **DNA2** and **DNA3**, L7 for the assembly of **Y-DNA** and **Ad-8**. **(B)** Tapping-mode AFM height and **(C)** TEM images of **Y-DNA/Ad-8** nanoparticles, respectively.

The synthesis of the 8-arm star PEG polymer, **Ad-8** (guest molecule) was achieved by following our previously reported procedure (Thelu et al., 2018). The β-CD-tethered oligonucleotides (**DNA1**–**3**) are complementary to each to form β-CD-tethered Y-shaped DNA (**Y-DNA**) and the sequences of the DNAs are provided in Table 1. The self-assembly of the oligonucleotides was initially studied using native polyacrylamide gel electrophoresis (PAGE) analyses (Figure 1 and Figure S1). The self-assembly of the β-CD-tethered Y-shaped DNA (host) was achieved by the self-assembly of equimolar quantities of **DNA1**–**3** in PBS buffer containing NaCl (100 mM) by annealing from 90°C to room temperature following a reported procedure (Um et al., 2006). Native PAGE analyses clearly show that the sequential addition of complementary DNAs leads to the formation of host β-CD-tethered Y-shaped DNA (**Y-DNA**) as it is evident from the reduced electrophoretic mobility of the resultant DNA nanostructure compared with the corresponding individual complementary DNA strands. Further reduction in the electrophoretic mobility was observed with the addition of **Ad-8** to **Y-DNA**, which clearly reveals that the self-assembly between **Ad-8** and **Y-DNA** through host-guest interaction between β-CD and adamantane resulted in the formation of nanoaggregates for **Ad-8/Y-DNA** supramolecular complex. Dynamic light scattering (DLS) analyses of **Ad-8/Y-DNA** show the formation of aggregated species in solution with diameter in the range of 140–290 nm. Zeta potential (ζ) measurement reveals a value of −16.6 mV, which suggest the formation of aggregated species decorated with negatively charged DNA. Better insights into the morphology of the aggregates were provided by atomic force microscopy (AFM) and transmission electron microscopy (TEM) analyses. Atomic force microscopic height image of **Y-DNA/Ad-8** shows the formation of spherical nanoparticle with diameters in the range of 150–300 nm. The section analyses of the nanoparticle reveal that the average height of the nanoparticle is ~18 nm, which is significantly lower than the corresponding average diameter of the nanoparticles. This indicates significant flattening of the nanoparticles occurs on the mica surface due to their soft nature, which is in accordance with similar soft nanoparticles. In accordance with AFM observations, TEM analyses also show the formation of nanoparticles with diameters in the range of ~250 nm. Microscopic analyses are in good agreement with the DLS data. Microscopic, DLS and PAGE data collectively confirm that hierarchical assembly of **Y-DNA** and **Ad-8** through multivalent host-guest interaction between β-CD and adamantane leads to the formation of **Y-DNA/Ad-8** nanoparticles in water (nanogel).

**Table 1:** Sequences of **DNA1–3**.

**DNA**	**Sequence (5′ → 3′)**
**DNA1**	β-CD-TTGGATCCGCATGACATTCGCCGTAAGA
**DNA2**	β-CD-TCTTACGGCGAATGACCGAATCAGCCTA
**DNA3**	β-CD-TAGGCTGATTCGGTTCATGCGGATCCAA

The most striking features of the nanoparticles include: (i) excellent biocompatibility, (ii) non-toxicity, (iii) hydrophobic pockets, and (iv) anionic DNA backbone, which are ideal for their application as an adsorbent for the capturing of hydrophobic and hydrophilic molecules dissolved in water. This motivated us to explore the potential of the DNA nanoparticles for the capturing of micropollutants typically present in water. We have taken few representative examples for hydrophilic and hydrophobic molecules to demonstrate the potential of the nanogel to capture them from water. Hydrophobic molecules investigated in this study include carcinogenic aromatic hydrocarbon such as perylene, anthracene, phenanthrene, and 1-naphthyl amine. The hydrophilic molecules selected in our study include calcein (organic dye) and propranolol hydrochloride (pharmaceutical). Typically, concentration of the micropollutants present in drinking water is in the range of nanomolar to micromolar concentrations. Hence, we have artificially contaminated the water with different micropollutants with a final concentration of 100 nM. Final concentration of DNA nanoparticles used in our study was 500 nM. Typically, **Y-DNA/Ad-8** nanoparticle in PBS buffer (100 μL) containing NaCl (100 mM) was added into water containing micropollutant solution (400 μL), shaken for 1 min and kept undisturbed for about 10 min. In the cases of hydrophobic micropollutants, they were initially dissolved in acetone and diluted with water to 400 μL. Since the concentration of the micropollutants present in all these cases is 100 nM, we have used very sensitive fluorescence spectroscopy as the tool to follow the capture of micropollutants. This was studied by comparing florescence intensity changes of the micropollutants before and after DNA nanoparticle addition to their solution.

**Figure 2:**
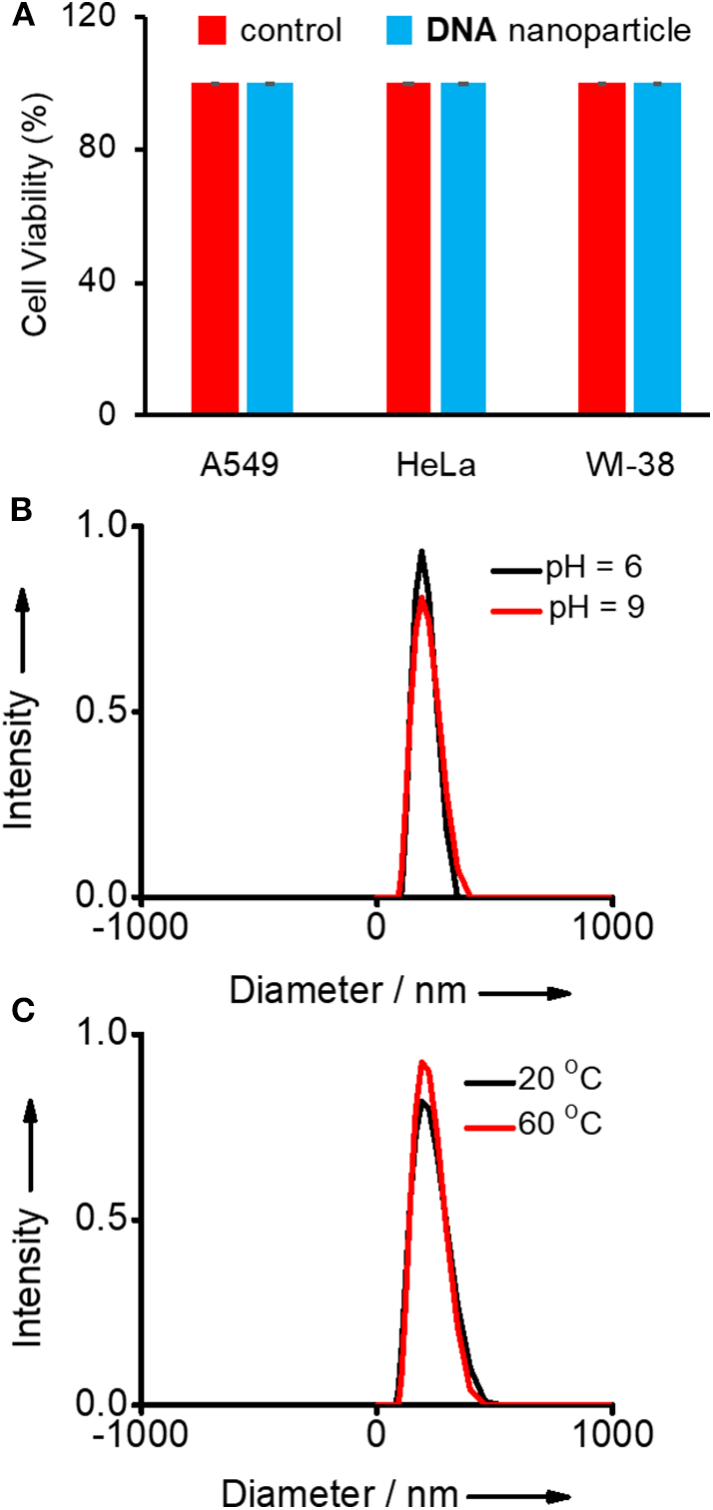
**(A)** Comparison of viability of A549, HeLa (cancer cells), and WI-38 (normal cell) cell lines treated with **Y-DNA**/**Ad-8** nanoparticles. Size distribution of nanoparticle obtained from the DLS analyses **(B)** at two different pH and **(C)** at two different temperatures.

Initially, the biocompatibility of the nanogel was analyzed with two different cancer cell lines (HeLa and A549) by means of 3-(4,5-dimethylthiazol-2-yl)-2,5-diphenyltetrazolium bromide (MTT) assay. The viability of untreated HeLa, A549, and WI-38 cells were taken as 100%. In order to assess the biocompatibility of **Y-DNA/Ad-8** nanoparticle, A549, HeLa (cancer cells), and WI-38 (normal cell) cells were incubated with **Y-DNA/Ad-8** nanoparticles for 24 h. The concentration of **Y-DNA/Ad-8** nanoparticle used for MTT assay was 500 nM. As expected, MTT assay reveals cell viability above 95% for all cell lines demonstrating excellent biocompatibility of **Y-DNA/Ad-8** nanoparticles (Figure 2A). Furthermore, thermal and pH stabilities of the nanoparticles under different experimental conditions were studied by DLS analyses. DLS analyses of the nanoparticle at pH 6 and 9 show no difference in the size distribution of the nanoparticle, which clearly show the pH stability of the nanoparticle (Figure 2B). Similarly, thermal stability of the nanoparticle was studied at 20 and 60°C and in this case also no noticeable change was observed for the size distribution of the nanoparticles (Figure 2C). These results reveal the thermal and pH stability of **Y-DNA/Ad-8** nanoparticles.

**Figure 3:**
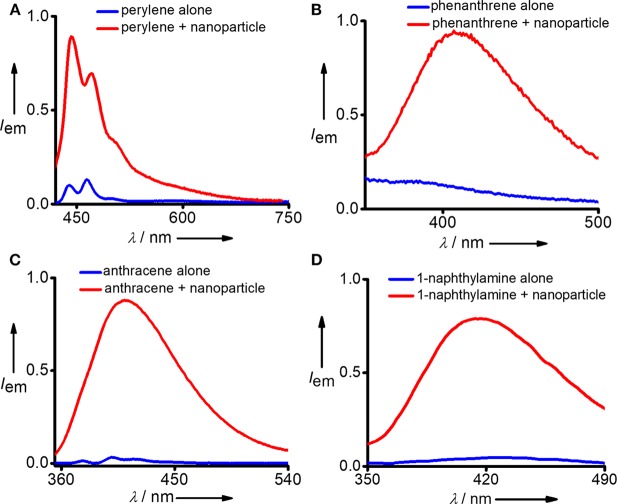
Comparison of fluorescence spectra of **(A)** perylene, **(B)** phenanthrene, **(C)** anthracene, and **(D)** 1-naphthylamine before and after the addition of **Y-DNA**/**Ad-8** nanoparticles.

After confirming the biocompatibility and stability of **Y-DNA/Ad-8** nanoparticles, we have carried out the micropollutant capture experiments with the nanoparticles. It is to be mentioned that the hydrophobic micropollutants selected in our study showed very poor solubility in water. Emission spectrum of perylene in water shows its characteristic emission with emission maximum centered at 440 nm (λ_exc_ = 400 nm) (Figure 3A and Figure S2). Very interestingly, a significant enhancement in emission intensity (9 fold) was observed with the addition of **Y-DNA/Ad-8** nanoparticles, which can be attributed to the capturing of feebly water-soluble perylene into the hydrophobic cavities of the nanoparticle. It is also to be noted that a red-shift of 3 nm was observed for the emission peak at 440 nm. These results collectively suggest that perylene binds efficiently to the nucleobases through π-stacking interactions as reported in similar systems (Sezi and Wagenknecht, 2013; Gershberg et al., 2015). The red-shift in the emission peak suggest that the perylene reside in the hydrophobic environments after their capture from water. The possibility of non-covalent trapping of perylene in the hydrophobic pockets of the nanoparticle cannot be completely ruled out. From the fluorescence titration experiments we have calculated the capture efficiency of the nanoparticle as 500 nM of **Y-DNA/Ad-8** nanoparticle was able to capture 1 μM of perylene. Anthracene was nearly insoluble in water as evident from its fluorescence spectrum (λ_exc_ = 400 nm) in water. However, efficient capturing of anthracene by the nanoparticles caused a dramatic increase in the fluorescence intensity (Figure 3B). In this case as well, π-stacking with the nucleobases and hydrophobic interactions are responsible for the capturing of anthracene by the DNA nanoparticles. It is also to be noted that the excimer emission with maximum centered at 410 nm dominates in the emission spectrum due to the aggregation of anthracene in the hydrophobic environment of the nanoparticle (Neelakandan and Ramaiah, 2008). Fluorescence spectrum of phenanthrene in water clearly reveals that it is nearly insoluble in water. However, a significant enhancement in emission intensity was observed with the addition of nanoparticle into the solution due to the efficient capture of phenanthrene by the nanoparticles (Figure 3C). It is also worth noting that phenanthrene shows mainly excimer-like emission centered at 408 nm (λ_exc_ = 280 nm) after their capture (Haynes et al., 1990), which clearly suggest that phenanthrene exists as aggregated species in the nanogel assembly. This observation is in good agreement with other hydrophobic molecules. We have also demonstrated the capture of 1-naphthylamine (Figure 3D). A dramatic increase in fluorescence intensity was observed with the capture of poorly water soluble 1-naphthylamine by the nanoparticles. In this case as well, only excimer-like red-shifted peak centered at 415 nm (λ_exc_ = 270 nm) was observed due to the aggregation of 1-naphthylamine in **Y-DNA/Ad-8** nanoparticle network.

**Figure 4:**
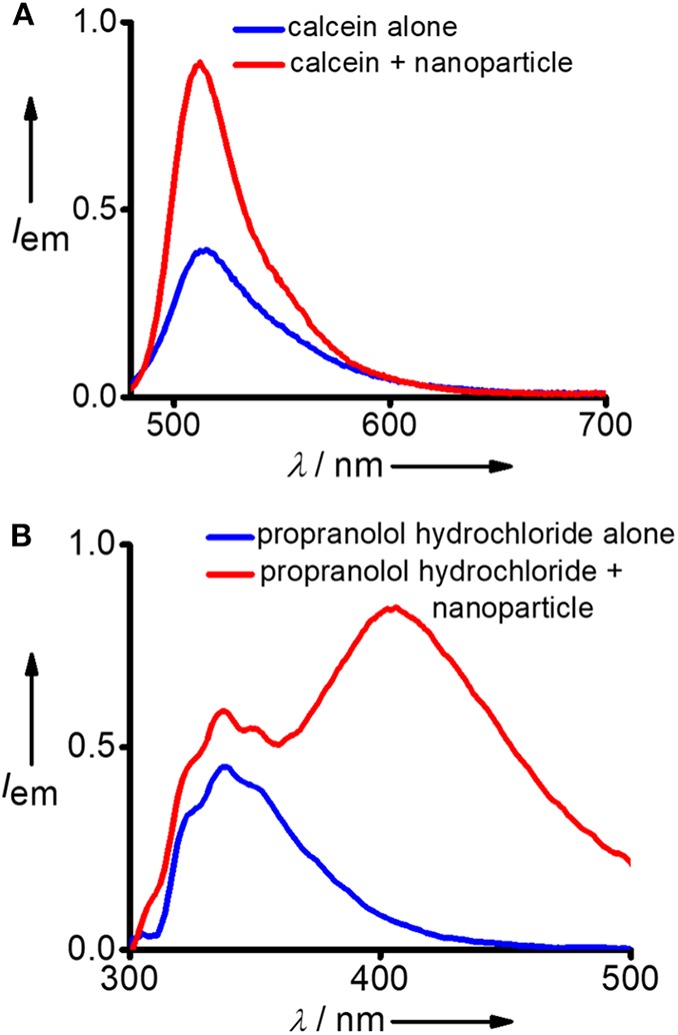
Comparison of fluorescence spectra of **(A)** calcein and **(B)** propranolol hydrochloride before and after the addition of **Y-DNA**/**Ad-8** nanoparticles.

After successful demonstration of the capture of hydrophobic micropollutants, we have extended our study toward the capture of hydrophilic micropollutants in water. We have taken calcein as a representative hydrophilic organic dye. Calcien is water soluble and it exhibits the characteristic emission spectrum with maximum centered at 512 nm (λ_exc_ = 470 nm). Interestingly, an increase in emission intensity was observed after the incubation with DNA nanoparticles (Figure 4A). This observation suggests that though calcien is water soluble, it is weakly aggregated in water and upon binding with the **Y-DNA/Ad-8** nanoparticle through non-covalent interactions dissociate the weakly assembled calcien aggregated species into the corresponding monomeric species, which resulted in an increase in emission intensity. Another hydrophilic molecule we have investigated in this study is propranolol hydrochloride, which is a pharmaceutical molecule. Propranolol hydrochloride is cationic molecule and is water soluble. This molecule shows its characteristic emission centered at 335 nm (λ_exc_ = 270 nm) in water (Figure 4B). Interestingly, in addition to the characteristic emission of propranolol hydrochloride at 335 nm, a new red-shifted peak centered at 405 nm also emerges after its incubation with **Y-DNA/Ad-8** nanoparticles. This suggest that positively charged propranolol hydrochloride efficiently binds to the negatively charged DNA backbone through electrostatic interactions with its hydrophobic naphthalene residue interact with the nucleobases through π-π stacking interactions. The emergence of red-shifted excimer-like peak suggest that the molecule is getting aggregated inside the nanogel network after their capture by the **Y-DNA/Ad-8** nanoparticles.

## Conclusions

In summary, we have reported the synthesis of physically cross-linked DNA nanoparticles through multivalent host-guest interaction between β-CD functionalized branched DNA nanostructures as the host and a star-shaped adamantyl-terminated 8-arm poly(ethylene glycol) polymer as the guest. The most remarkable structural features of DNA nanoparticles include their excellent biocompatibility and the possibility of various non-covalent interactions with both hydrophobic and hydrophilic organic molecules. We have demonstrated the potential of DNA nanoparticles for the rapid and efficient capture of various micropollutants typically present in water including carcinogens (hydrophobic micropollutants), organic dyes (hydrophilic), and pharmaceutical molecules (hydrophilic). The capture of micropollutants by DNA nanoparticles was attributed to the various non-covalent interactions between DNA nanoparticles and the micropollutants. The excellent biocompatibility of the DNA nanoparticles and their potential as an efficient capture of both poorly water soluble hydrophobic micropollutants and highly water soluble hydrophilic micropollutants may motivate other researches to explore DNA-based nanomaterials as an adsorbent for the removal and purification of micropollutants from drinking water.

## Data Availability Statement

All datasets generated for this study are included in the article/Supplementary Material.

## Author Contributions

RV designed the project. SA, HT, DP, and KH performed all the experiments. RV and SA co-wrote the manuscript. All authors analyzed the data and commented on the manuscript.

### Conflict of Interest

The authors declare that the research was conducted in the absence of any commercial or financial relationships that could be construed as a potential conflict of interest.
