# Supplementary material for: Efficient Capturing of Polycyclic Aromatic Micropollutants From Water Using Physically Crosslinked DNA Nanoparticles
Source: Front Chem. 2020 Jan 28;8:2. doi: 10.3389/fchem.2020.00002 (PMC6999083; doi:10.3389/fchem.2020.00002)
Supplement: Supplementary file 1 [file Table_1.docx]

**Supporting Information**

***for***

**Efficient capturing of polycyclic aromatic micropollutants from water using physically crosslinked DNA nanoparticles**

Siriki Atchimnaidu, Hari Veera Prasad Thelu, Devanathan Perumal,

Kaloor S. Harikrishnan, Reji Varghese*

Indian Institute of Science Education and Research Thiruvananthapuram (IISER-TVM)

Trivandrum-695551, Kerala, INDIA

Email: [reji@iisertvm.ac.in](mailto:reji@iisertvm.ac.in)

|  | **TABLE OF CONTENTS** |  |
| --- | --- | --- |
| **SI. No.** | **Contents** | **Pages** |
| 1 | Experimental section | 02-03 |
| 2 | Comparison of fluorescence studies of Y-DNA, perylene and nanoparticles | 03-03 |
| 3 | Gel electrophoresis of Y-DNA and alcohol functionalized polymer | 04-04 |

**Experimental section**

All chemicals used for the experiments were purchased from Sigma Aldrich and were used as received. Alkyne modified phosphoramidites for automated solid phase DNA synthesis were purchased from Glen Research. TLC analyses were performed on aluminium plates coated with silica gel 60 F254, and column chromatography was performed on 200– 400 mesh silica gel. ^1^H and ^13^C NMR spectra were recorded on a Bruker Avance 500 MHz spectrometer using 1,1,1,1-tetramethylsilane (TMS) as an internal standard. The water used for all studies was Milli-Q deionised water (18.2 MΩ cm). Mass measurements were performed on a Shimadzu GC-MS, QP-2010. HR-MS analyses were performed on a Waters Xevo G2 QTof LC/MS system. Measurements were done in negative ion mode. Oligonucleotides were synthesised on a H-8 K&A DNA synthesizer. The alkyne-modified oligonucleotide was purified on a reverse column (Phenomenex Luna C-18 column) on a Shimadzu HPLC system. MALDI-TOF analysis were carried on Bruker UltrafleXtreme MALDI-TOF spectrometer. Native PAGE analyses were performed on an SCIE–PLAS TV400YK vertical electrophoresis unit. Ethidium bromide stained gels were imaged using a Syngene Chemi XR5 gel documentation system. AFM analyses were carried out on a Multimode SPM (Veeco Nanoscope V). Samples were prepared by drop-casting 5 μL solution of the sample on a freshly cleaved mica surface and dried under air. The probe used for imaging was an antimony doped silicon cantilever with a resonant frequency of 300 kHz and a spring constant of 40 Nm^-1^. TEM analyses were carried out on an FEI Tecnai G2 F20 (200 kV) high-resolution TEM. Samples were prepared by depositing 2 μL of the sample on a 400 mesh carbon coated copper grid (Ted Pella, Inc.) which was negatively glow discharged (PELCO easiGlow, glow discharge cleaning system) for 1 min prior to use. The samples were allowed to adsorb on the grid for 2 min and then excess sample was wicked with a piece of filter paper and the sample was stained with 0.7% uranyl formate stain solution. Absorption spectra were recorded using a quartz cuvette of 10 mm path length on a Shimadzu UV-3600 Vis-NIR Spectrophotometer having a Peltier controlled cell holder. Steady state fluorescence spectra were recorded on a Horiba Jobin Yvon Fluorimeter equipped with a thermostat Peltier cell holder, in a quartz cuvette of 10 mm path length. Cell viability has been determined by means of MTT assay. Cell lines (A549 and HeLa) were borrowed from NCCS, Pune. DMEM, RPMI, antibiotic solution 100X liquid-1000U penicillin and 10 mg streptomycin per mL were obtained from Hi-media Lab Pvt Ltd, India, and 10% FBS was obtained from PAN-Biotech GmbH, Germany. HeLa and A549 cells were seeded at the density of 1 × 10^4^ cells/well in a 96 well culture plate (Corning) with DMEM and RPMI culture medium respectively and grown in 5% CO_2_ incubator at 37 ºC for 24 h. The cells were incubated with and without DNA nanoparticles in separate wells along with fresh media. After 12 h incubation, the culture medium was removed and add fresh culture medium and kept for further cell growth for 48 h in 5% CO_2_ incubator at 37 ºC. Cytotoxicity was analysed by MTT assay. 10 μL of 12 mM MTT (3-[4, 5-dimethylthiazol-2-yl]-2, 5- diphenyltetrazolium bromide) was added and kept for 4 h incubation at 37 ºC in 5% CO_2_ incubator. 150 μL DMSO was then added to solubilize the formazan crystals formed and absorbance was measured at 565 nm using Tecan Infinite 200 PRO microplate reader.

**Figure S1**. Comparison of fluorescence spectra of Y-DNA, perylene, perylene & Y-DNA and perylene & nanoparticle. Fluorescence spectra was recorded at an excitation wavelength of 400 nm.


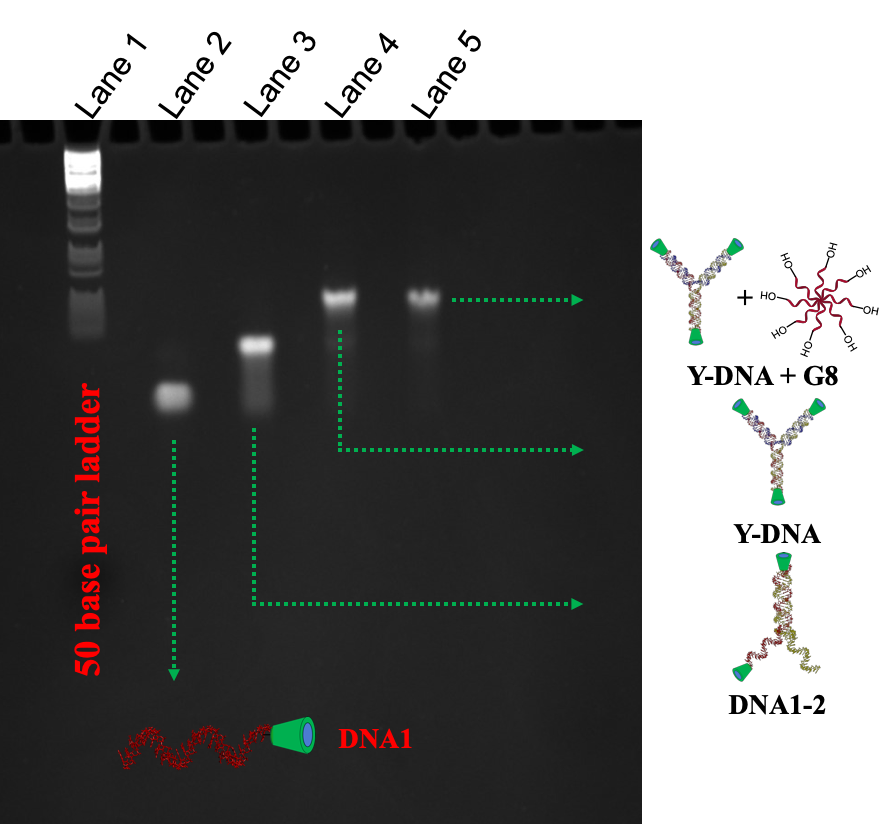


**Figure S2**. Native PAGE (14%) analyses of the self-assembly of **Y-DNA/G-8**. **G-8** corresponds to alcohol terminated 8-arm star PEG polymer, which lacks adamantane moiety. Lane 1 for 50 base pair ladder, Lane 2 for **DNA1**, Lane 3 for **DNA1+DNA2**, Lane 4 for **Y-DNA**. Lane 5 for **Y-DNA+G-8**.
